# The application of tubeless anesthesia technique in tracheal tumor resection surgery: a case report

**DOI:** 10.1097/MS9.0000000000005657

**Published:** 2026-09-09

**Authors:** Jing-Ting Tang, Huilu Li, Bo Wu, Qian Liu, Ju-Ke Ma

**Affiliations:** a Department of Anesthesiology, Shandong Second Provincial General Hospital, Jinan, China; b Department of Head and Neck Surgery, Shandong Second Provincial General Hospital, Jinan, China; c Department of Pathology, Shandong Second Provincial General Hospital, Jinan, China

**Keywords:** hypopharyngeal carcinoma, hemangioma, neoadjuvant immunotherapy, no-intubation anesthesia, total laryngectomy

## Abstract

Systemic hemangioma after neoadjuvant immunotherapy for hypopharyngeal carcinoma is one of the common complications of neoadjuvant immunotherapy. Its pathogenesis may be related to multiple immune regulatory imbalances and vascular biological changes. This case was a locally advanced hypopharyngeal cancer patient who received neoadjuvant immunotherapy and total laryngectomy with partial hypopharyngectomy. During follow-up, a mass was found in the middle part of the trachea, protruding into the tracheal lumen. The peculiarity of this case was that the tracheal mass was located in the middle part of the trachea, with a narrow lumen. During anesthesia, whether the tracheal intubation was placed above or below the mass would affect the operation. After multidisciplinary team discussion, a no-intubation anesthesia plan was adopted, using esketamine, which could maintain spontaneous breathing well, ensure sufficient depth of anesthesia, and reduce the occurrence of bronchospasm. The airway management mainly used high-flow nasal cannula to provide a large flow and flow rate, prolonging the safe period without breathing and oxygenation, providing a safety guarantee for the operation and ensuring its successful completion.

## Introduction

Non-intubated anesthesia with spontaneous breathing has been previously utilized in thoracoscopic surgery, as it eliminates the need for tracheal intubation and mechanical ventilation^[^1^]^. This approach effectively reduces airway trauma and associated pulmonary complications, improves patient outcomes, shortens hospital length of stay, and facilitates the achievement of enhanced recovery after surgery goals. In recent years, high-flow nasal cannula (HFNC) has been increasingly adopted in diagnostic and therapeutic procedures involving rigid bronchoscopy and airway management under general anesthesia, particularly in otorhinolaryngological surgeries^[^2^]^, where it has demonstrated notable advantages. Otorhinolaryngological procedures frequently involve the head and neck region, often present with difficult airways, and require sharing the airway with the surgical team during the operation. Consequently, these scenarios impose higher demands on anesthesiologists’ airway management expertise, necessitating mastery of and flexible application of innovative airway techniques to ensure patient safety, smooth perioperative transition, and high-quality postoperative recovery. The HFNC system comprises an air-oxygen mixing device, a heated humidification module, and a unidirectional inspiratory circuit. It enables precise control of inspired oxygen concentration and gas flow rate, while maintaining optimal temperature and humidity of the delivered gas, thereby enhancing patient comfort and adherence to oxygen therapy.

HIGHLIGHTS

- Surgical resection was successfully performed for a neoadjuvant immunotherapy-induced tracheal hemangioma with airway obstruction in a post-total-laryngectomy hypopharyngeal carcinoma patient.
- The preoperative multidisciplinary team evaluation excluded an indication for ECMO and formulated personalized anesthesia strategies and emergency contingency plans.
- Tubeless anesthesia was adopted for lesion excision, achieving stable perioperative respiratory and circulatory homeostasis.
- The favorable prognosis provides a clinical reference for complex airway management in specific laryngectomy populations.

Systemic hemangioma following neoadjuvant immunotherapy for hypopharyngeal cancer is a recognized complication associated with this treatment modality. Evidence suggests that its development is linked to excessive immune system activation and autoimmune responses, cytokine storms accompanied by the release of angiogenic factors, dysregulation of vascular endothelial cell signaling pathways, and genetic predisposition and environmental influences^[^3–5^]^. This report describes a case of locally advanced hypopharyngeal cancer in which a tracheal mass was identified during follow-up after neoadjuvant therapy, partial hypopharyngectomy, and total laryngectomy. The patient subsequently underwent tracheal resection and formation of an anterior cervical fistula. The case presented unique clinical challenges. Following a multidisciplinary team (MDT) discussion, a modified anesthetic approach was implemented, involving spontaneous breathing without endotracheal intubation, supplemented with HFNC support. The anesthetic and surgical procedures proceeded uneventfully, and the overall treatment outcome was favorable. This case report was written in accordance with the SCARE 2025 reporting guidelines^[^6^]^. Artificial intelligence tools were used only for grammatical revision and language polishing of the manuscript text. No AI software participated in case data collection, surgical analysis, or manuscript content generation.

## Case presentation

A 68-year-old male patient was admitted to our hospital with the chief complaint of hemoptysis for 1 week, occurring 1 year after hypopharyngeal cancer surgery. One year prior to this admission, the patient had presented to our department with dysphagia. Initial physical examination revealed a cauliflower-like mass on the posterior wall of the hypopharynx, and pathological biopsy confirmed hypopharyngeal squamous cell carcinoma (T3N2M0, involving the posterior wall). The patient subsequently received two cycles of neoadjuvant immunotherapy with pembrolizumab combined with albumin-bound paclitaxel, and post-treatment clinical evaluation indicated a partial response. Following an MDT discussion, surgical intervention was recommended. In March 2024, the patient underwent bilateral cervical lymph node dissection, total laryngectomy, and hypopharyngectomy under general anesthesia (Fig. 1E). Postoperatively, he completed concurrent chemoradiotherapy without significant complications, and the entire treatment course was uneventful. He was then discharged and had been attending regular follow-up visits until the current admission.

**Figure 1.**
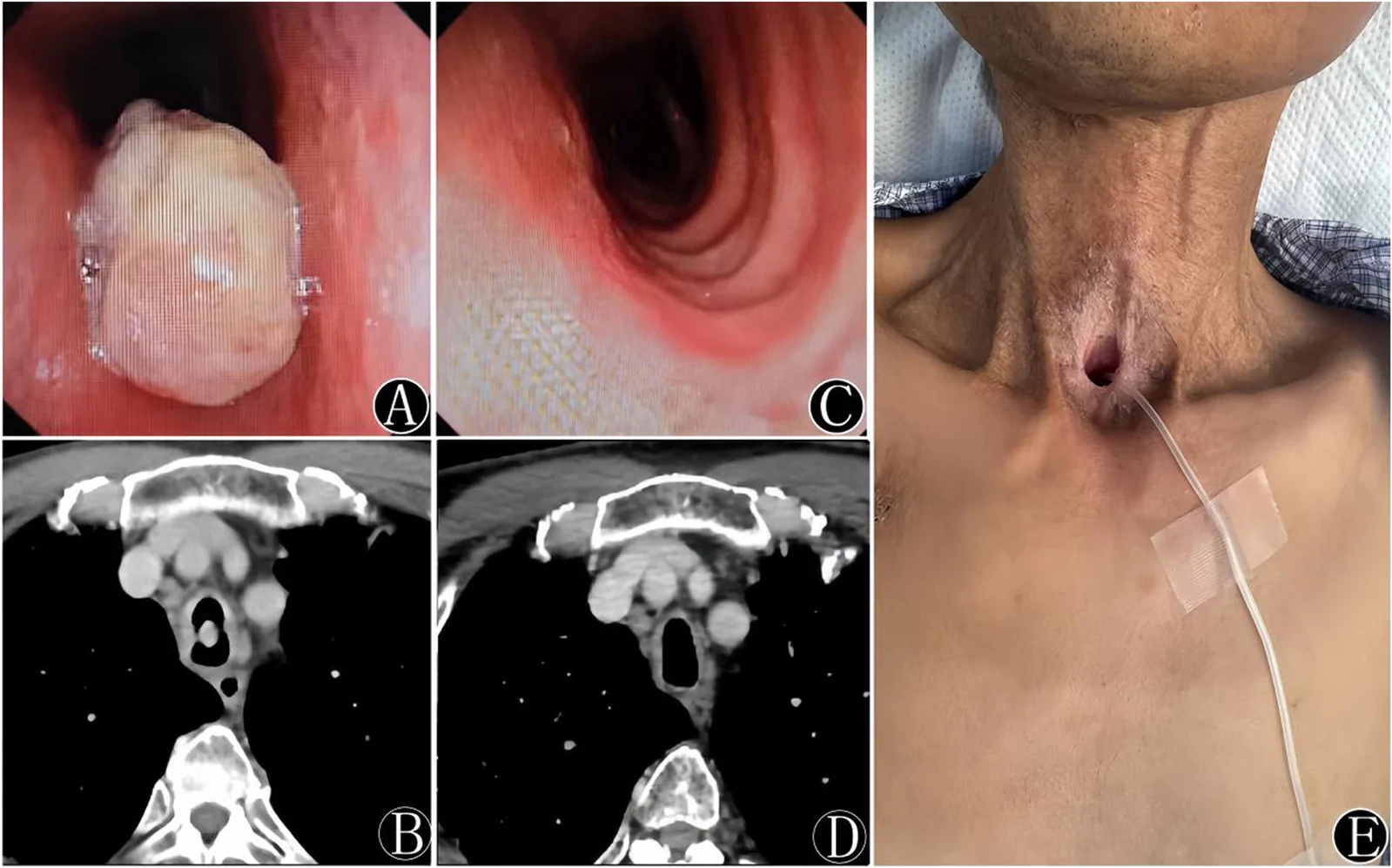
Imaging and intraoperative findings of tracheal tumor resection in a patient with hypopharyngeal cancer, status post-total laryngectomy. (A) Electronic laryngoscopy showing a protruding mass in the mid-trachea with an irregular surface extending into the tracheal lumen; (B) Contrast-enhanced chest CT demonstrating a soft-tissue mass in the mid-trachea at the level of the aortic arch with marked enhancement, consistent with a pedunculated lesion attached to the right tracheal wall; (C) Electronic laryngoscopy at 1-month follow-up showing smooth tracheal mucosa without residual or recurrent tumor; (D) Contrast-enhanced chest CT at 1-month follow-up showing no evidence of a soft-tissue mass in the trachea; (E) Intraoperative photograph of the patient (status post-total laryngectomy for hypopharyngeal cancer) with a cervical tracheostomy; high-flow oxygen was administered via the tracheostomy to maintain spontaneous breathing during the uneventful surgical procedure.

One week prior to the present hospitalization, the patient developed hemoptysis and dyspnea without an identifiable trigger. On admission, electronic laryngoscopy revealed a pale-red, protruding mass with an irregular surface on the right side of the trachea, which protruded into the tracheal lumen and occupied approximately 45% of the airway diameter (Fig. 1A). Chest computed tomography (CT) demonstrated a soft-tissue mass located about 6 cm below the tracheostomy site, extending approximately 5 cm proximal to the carina (predominantly in the mid-trachea between the tracheostomy and carina). The mass showed marked enhancement on contrast-enhanced CT, suggestive of a rich blood supply (Fig. 1B). The maximum tracheal diameter was measured at approximately 2.28 cm, and cross-sectional images revealed that the mass completely occluded the tracheal lumen in some views, posing substantial challenges for endotracheal intubation. A preoperative multidisciplinary consultation concluded that endotracheal tube placement in the tracheal segment above the mass would interfere with the planned surgical procedure. Even if the tube were placed across the mass for deep anesthesia, it would still compromise the surgical field of view and operative space, thereby increasing surgical risk and difficulty. Therefore, it was unanimously decided to perform tubeless general anesthesia for the procedure.

Preoperative laboratory tests, including complete blood count, electrolytes, and liver and renal function, showed no significant abnormalities. Contrast-enhanced neck and chest CT revealed loss of normal pharyngeal and laryngeal anatomical structures, a soft tissue mass on the right side of the mid-trachea, and disease progression compared with prior imaging. Electrocardiography indicated a normal sinus rhythm, and echocardiography showed mild mitral regurgitation with a left ventricular ejection fraction (EF) of 0.61%. Preoperative anesthetic assessment classified the patient as ASA Grade II. Airway obstruction due to the mass was graded as Myer–Cotton Grade II (airway stenosis of 51%–70%). Physical examination revealed slightly diminished bilateral breath sounds, with crackles auscultated in the lower lung lobes; the patient had normal breathing at rest but experienced dyspnea with activity or when the airway was obstructed by dry sputum crusts, without inspiratory retractions (suprasternal, intercostal, or subcostal). In the operating room, the patient was placed in the supine position, and standard monitors were applied, showing the following baseline vital signs: heart rate (HR) 77 beats/min, blood pressure (BP) 135/77 mmHg, respiratory rate (RR) 13 breaths/min, pulse oxygen saturation (SpO_2_) 98%, and Bispectral Index (BIS) 96, with no obvious dyspnea. Oxygen was administered via the tracheostomy at 100% fraction of inspired oxygen (FiO_2_) and a flow rate of 8 L/min. Dexmedetomidine was administered intravenously at a rate of 2.5 μg/kg/h, and lidocaine aerosol (Likituo, Xiangxue Pharmaceutical) was administered for topical tracheal anesthesia via the tracheostomy every 5 minutes, repeated three times. Simultaneously, a breathing circuit was connected to a disposable reinforced tracheal tube (Haiseng Medical, Model RTI-5.5A). The proximal end of the tracheal tube was connected to the distal interface of a disposable intravenous infusion needle (Jierei, 0.7 × 19 TWLB); after removing the needle component, the infusion extension tube was advanced 3 cm into the trachea via the tracheostomy to establish an oxygen delivery route. The FiO_2_ was maintained at 100%, and the oxygen flow rate was adjusted to 5 L/min. Ten minutes later, the dexmedetomidine infusion was discontinued, at which point the patient’s Ramsay Sedation Scale (RSS) score was 4–5. Anesthesia induction was then performed with sequential intravenous administration of atropine 0.5 mg, palonosetron 0.25 mg, dexamethasone 10 mg, remimazolam 15 mg, and alfentanil 0.25 mg, followed by continuous intravenous infusions of remimazolam (0.5–2 mg/kg/h) and remifentanil (5–20 μg/kg/h). The surgical procedure commenced when the BIS value decreased to 60 ± 2. A 0° nasal endoscope was inserted via the tracheostomy for visualization. Intraoperative inspection revealed a pedunculated mass attached to the right tracheal wall. Bipolar electrocoagulation was used to incise the mass at its base and achieve hemostasis simultaneously. The mass was successfully resected with an estimated blood loss of approximately 1 mL. After complete tumor resection, bipolar electrocoagulation was re-applied to the base for hemostasis reinforcement. The entire surgical procedure was completed successfully in 13 minutes. Intraoperatively, the infusion rates of remimazolam and remifentanil were dynamically adjusted based on real-time BIS monitoring to maintain the BIS value within the target range of 50–65. Vital signs were continuously monitored to ensure the depth of anesthesia was adequate for the surgical procedure. The patient maintained good spontaneous breathing throughout the operation, without coughing, dyspnea, or body movement. SpO_2_ was sustained at 95%–98%, and no significant fluctuations in BP or HR were observed. Arterial blood gas (ABG) analysis was performed immediately after surgery, showing a partial pressure of carbon dioxide (PaCO_2_) of 55 mmHg, indicative of mild hypercapnia. A repeat ABG analysis 10 minutes later showed that PaCO_2_ had decreased to 42 mmHg, returning to the normal range. Flumazenil 0.3 mg was then administered intravenously, and the patient opened his eyes 2 minutes later, regained full consciousness, and reported no specific discomfort. After discontinuation of oxygen therapy, SpO_2_ remained at 95%–98%, and the patient was transferred to the general ward in a stable condition.

Postoperatively, the patient received daily nebulization therapy and empirical anti-infective treatment. He did not report dyspnea or hemoptysis during the postoperative period. The patient was discharged on the fifth postoperative day and has since had an uneventful follow-up with a favorable prognosis.

## Discussion

This case involves a patient who developed progressive dyspnea following total laryngectomy for hypopharyngeal cancer, which was preceded by neoadjuvant immunotherapy. On admission, electronic laryngoscopy revealed a large intratracheal mass with an irregular surface protruding into the tracheal lumen, and the pathological nature of the mass was initially undetermined. When considered alongside findings from contrast-enhanced chest CT, the clinical presentation was initially suspicious for mediastinal or pulmonary metastasis following chemoradiotherapy for hypopharyngeal cancer. To obtain a definitive histopathological diagnosis and guide subsequent management, surgical biopsy and resection were deemed necessary. However, the patient’s prior total laryngectomy and permanent cervical tracheostomy presented significant challenges to performing the procedure under conventional intubated general anesthesia. First, the tracheostomy stoma was relatively narrow, and endotracheal tube placement – either above or below the intraluminal mass–would occupy critical tracheal space, precluding safe surgical access and manipulation. Second, intraoperative hemorrhage from the highly vascular mass was a major concern, as blood spillage into the distal respiratory tract could lead to acute dyspnea or even life-threatening asphyxia. To mitigate this risk, a flexible bronchoscope was prepared preoperatively for emergency endotracheal intubation (to occlude the airway distal to the bleeding site) and bronchoscopic suctioning (to clear blood from the distal airways and prevent obstruction) if needed.

Accordingly, a comprehensive anesthetic and surgical strategy, along with detailed contingency plans for potential intraoperative complications, was meticulously formulated in a preoperative multidisciplinary meeting. The primary challenge in anesthetic management was to maintain adequate oxygenation and ventilation, suppress airway reflexes during the procedure, and ensure rapid response to emergent events to safeguard patient safety and procedural success. For these reasons, an MDT consultation involving head and neck surgeons, anesthesiologists, and radiologists was held, and it was unanimously agreed to proceed with tubeless general anesthesia with spontaneous breathing during the procedure. Additionally, given the high airway reactivity associated with intratracheal surgery and the risk of hemorrhage, the team emphasized that, in addition to minimizing surgical duration, strict control of the anesthetic course and maintenance of adequate anesthetic depth were crucial to ensure sufficient oxygen supply and prevent adverse airway events.

According to the Myer–Cotton classification system for airway stenosis, the patient’s airway obstruction was approximately 65%, corresponding to Grade II stenosis^[^7,8^]^, defined as luminal obstruction of 51%–70%, with dyspnea on exertion but no inspiratory retractions. This case differs from conventional tubeless anesthesia cases in that the patient had previously undergone total laryngectomy, leaving a residual tracheostoma on the anterior neck postoperatively, and the tracheal mass was located in the mid-segment of the remaining trachea, with a narrow lumen. Endotracheal tube placement either above or below the mass would have compromised surgical access and visualization. Furthermore, the patient was elderly, with a long smoking history, impaired pulmonary function, reduced oxygen reserve, and incompletely recovered cardiopulmonary function (only 2 months after total laryngectomy and neoadjuvant immunotherapy for hypopharyngeal cancer). These factors necessitated careful selection of anesthetic drugs and individualized anesthetic planning to avoid an intraoperative oxygen supply–demand imbalance.

Pre-anesthetic preparation included adequate sedation and high-flow oxygen inhalation to optimize oxygen reserves. Anesthesia induction was performed slowly and smoothly to avoid respiratory depression induced by sedative and analgesic drugs, which could have exacerbated a difficult airway. Maintenance of adequate anesthetic depth was critical to prevent coughing, airway spasm, and hypoxemia during tracheal mass resection. The choice of ventilation mode was another key consideration: it needed to ensure adequate patient ventilation while providing an unobstructed surgical field for the operating surgeon^[^9^]^. We elected to use tubeless general anesthesia with spontaneous breathing and assisted mechanical ventilation, a strategy that offered several advantages. Assisted mechanical ventilation can significantly extend the safe window for impaired or insufficient spontaneous ventilation, prolong the period of adequate oxygenation without spontaneous breathing, and exert an effect similar to that of continuous positive airway pressure^[^10^]^. This approach effectively increases alveolar oxygen concentration, rapidly ameliorates hypoxic symptoms, prevents diaphragmatic fatigue, and provides a critical safety margin for the surgery. It also reduces the need for forced intraoperative interruptions and allows for a longer effective surgical duration.

Intraoperative ventilation parameters were tailored to the patient’s condition: low tidal volumes were used to minimize the potential for gas-induced displacement of the mass, and a high respiratory rate was employed to optimize oxygen diffusion into the alveoli and maintain oxygenation. Pure oxygen (100% FiO_2_) was administered throughout the procedure, and energy-based devices with a risk of airway thermal injury (e.g., monopolar electrocautery, laser) were avoided; bipolar electrocoagulation was used instead for resection and hemostasis due to its lower risk of airway damage. Intraoperative hemorrhage management protocols included the use of epinephrine-soaked cotton balls for compressive hemostasis and prompt suctioning of blood to prevent airway obstruction. In the event of massive hemorrhage, the contingency plan was to immediately place the patient in a head-down position, perform emergency endotracheal intubation, and position the tracheal tube cuff above the bleeding site to seal the distal trachea and protect the airway.

The surgical technique was optimized for the tubeless anesthetic approach: an assistant provided visualization via a rigid 4 mm, 0° nasal telescope, while the surgeon gently grasped the tumor with one hand and used bipolar electrocoagulation with the other to coagulate and incise the tumor’s pedicle, achieving simultaneous tumor resection and hemostasis. The procedure was completed uneventfully in just 13 minutes. The patient remained hemodynamically stable throughout the operation, with no coughing, choking, or adverse airway events. Dexamethasone, 10 mg, was administered intraoperatively to prevent tracheal edema. Intraoperative frozen-section pathology of the resected specimen confirmed a tracheal hemangioma (Fig. 2), a benign vascular lesion that explained the marked enhancement on contrast-enhanced CT and the risk of hemorrhage. The patient regained consciousness rapidly postoperatively with no reported discomfort, coughing, or dyspnea, and was safely transferred to the ward. Perioperative management included routine corticosteroid nebulization therapy to reduce airway edema, and the patient’s tracheal wound healed uneventfully with re-epithelialization of the mucosal surface. Regular follow-up with electronic laryngoscopy and cervical CT was conducted for 24 months after surgery. No evidence of hemangioma recurrence or tracheal scar hyperplasia was observed, and the patient’s respiratory function remained stable (Fig. 1 C,D).

**Figure 2.**
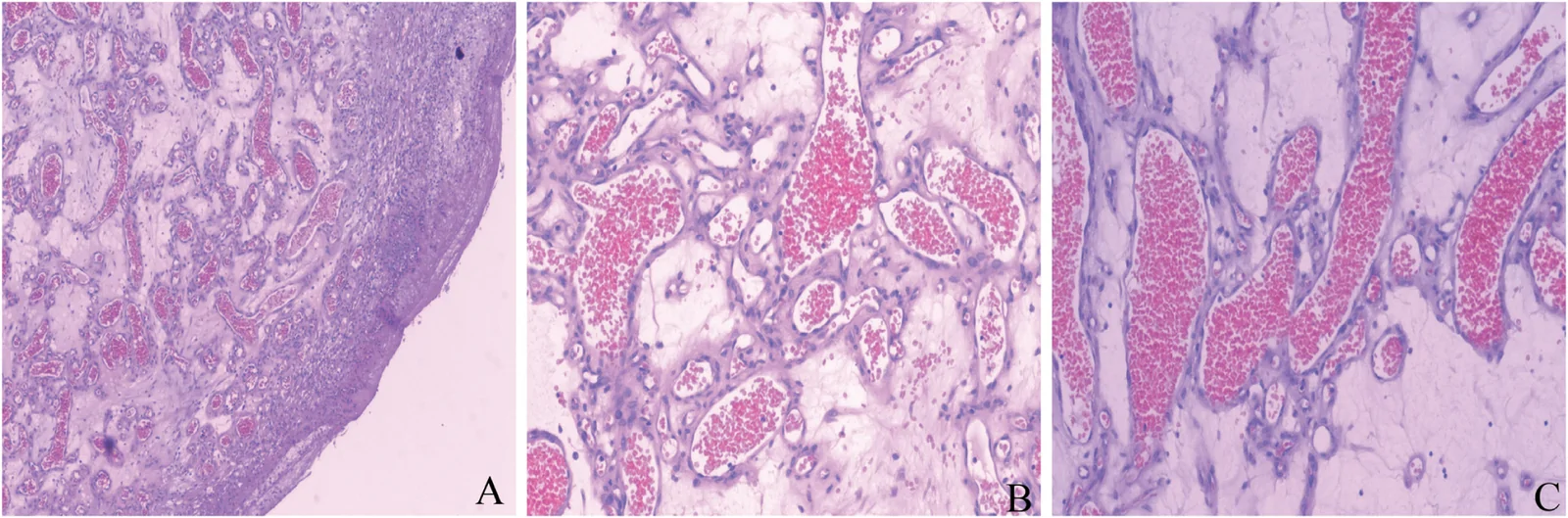
The postoperative pathological result of the tracheal mass was confirmed to be a hemangioma. (A) The mass is composed of proliferated blood vessels, with vascular lumina that are distended and filled with blood, accompanied by superficial ulceration and tissue disintegration (HE, 40×). (B) The lesion consists of proliferated blood vessels of varying luminal caliber with thin walls, and the vascular lumina are engorged with blood (HE, 100×). (C) The lesion consists of proliferated blood vessels lined by thin walls. The overlying squamous epithelium exhibits hyperplasia, hypertrophy, and parakeratotic changes, consistent with a hemangioma (HE, 100×).

## Conclusions

This case report describes the successful management of a patient with tracheal hemangioma-induced airway obstruction following neoadjuvant immunotherapy and total laryngectomy for hypopharyngeal cancer. A low-complexity diagnostic biopsy was initially planned, and extracorporeal membrane oxygenation (ECMO) was not deemed necessary during preoperative assessment. Following a comprehensive evaluation of the airway anatomy and potential intraoperative risks – conducted through MDT deliberation – a meticulous anesthesia strategy and robust emergency contingency plan were developed. The diagnostic procedure was successfully performed under non-intubated, spontaneously breathing anesthesia. The patient maintained hemodynamic and respiratory stability throughout the perioperative period, and the clinical outcome was favorable. We anticipate that this case report will serve as a valuable reference for the clinical management of analogous airway challenges.

## Data Availability

The datasets used and analyzed during the current study are available from the corresponding author upon reasonable request. Patient clinical information cannot be shared publicly to protect individual privacy.

## Research registration unique identifying number (UIN)

This case report was not prospectively registered because it is a single retrospective clinical case.
